# Editorial: Impulsivity and compulsivity related to substance use disorders

**DOI:** 10.3389/fpsyt.2025.1599890

**Published:** 2025-04-22

**Authors:** Francisca López-Torrecillas

**Affiliations:** Center Research Mind Brain and Behavior (CIMCYC), University of Granada, Granada, Spain

**Keywords:** impulsivity, compulsivity, substance use disorder, psychiatric disorders, inhibitory control

Impulsivity and compulsivity are fundamental behavioral constructs controlled by brain mechanisms essential for survival across species. When these mechanisms become dysfunctional, they contribute to a wide range of psychiatric disorders, imposing significant personal, social, and economic burdens. Understanding the neural underpinnings of impulsivity and compulsivity can facilitate targeted treatment strategies for individuals suffering from these maladaptive behaviors.

The American Psychiatric Association (APA, 2013) defines impulsivity as a predisposition toward rapid, unplanned reactions to internal or external stimuli, often disregarding negative consequences. Compulsivity, on the other hand, is characterized by repetitive behaviors aimed at reducing or preventing anxiety or distress, rather than seeking pleasure or gratification. Despite their differences, both constructs involve disruptions in response control and are mediated by overlapping yet distinct neural circuits, particularly those associated with motivational and decisional processes within the basal ganglia, limbic cortical inputs, and prefrontal control networks. While compulsive disorders such as obsessive-compulsive disorder (OCD) are often linked to increased frontal lobe activity, impulsive disorders like substance use disorders (SUD) and antisocial personality disorder (APD) are associated with reduced frontal lobe function.

Recent research has highlighted the role of impulsivity and compulsivity as risk factors in substance use disorders. Substance Use Disorder (SUD) is characterized by dysregulation in reward processing and inhibitory control systems, leading to increased sensitivity to immediate rewards and impaired response inhibition. Inhibitory control deficits have been recognized as both a determinant and a consequence of substance use disorders. This impaired inhibitory control results in difficulty resisting urges to consume substances despite negative consequences, further perpetuating the cycle of addiction. Additionally, impulsivity has been associated with higher rates of treatment dropout and relapse, emphasizing the need for interventions that specifically target this dimension.

The relationship between impulsivity, compulsivity, and psychiatric conditions remains a topic of debate. Some studies suggest that these constructs are distinct, with impulsivity being strongly linked to Substance Use Disorder (SUD) and aggressive behaviors, whereas compulsivity has been predominantly studied in the context of OCD. However, others argue that these constructs overlap, particularly in disorders that transition from impulsive goal-directed behavior to compulsive stimulus-driven behavior, such as addiction and obsessive-compulsive spectrum disorders. Given their commonalities and differences, impulsivity and compulsivity are often used interchangeably to describe self-control deficits that contribute to repetitive psychopathological behaviors. This raises important questions about how these constructs should be conceptualized and measured, as well as the implications for diagnosis and treatment.

In addition to their role in substance use disorders, impulsivity and compulsivity are also central to other psychiatric conditions, including attention-deficit/hyperactivity disorder (ADHD), borderline personality disorder (BPD), and eating disorders. For instance, individuals with ADHD frequently display elevated levels of impulsivity, which can manifest as difficulty delaying gratification, poor decision-making, and heightened risk-taking behaviors. Meanwhile, BPD is characterized by both impulsive and compulsive tendencies, with individuals exhibiting impulsive behaviors such as self-harm and compulsive behaviors like repetitive reassurance-seeking. Similarly, in eating disorders, impulsivity has been linked to binge-eating episodes, whereas compulsivity is associated with rigid dietary restrictions and obsessive thoughts about food and body image.

This Research Topic aims to differentiate impulsivity and compulsivity, providing insight into their unique contributions to psychiatric disorders, particularly in the context of addictive disorders and maladaptive behaviors. The included studies explore various aspects of these constructs, offering valuable perspectives on their underlying mechanisms and clinical implications:

Hinuma et al. classify patterns of intrusive thoughts based on their mechanisms of emergence and persistence, contributing to a better understanding of cognitive factors underlying compulsivity. Intrusive thoughts are a hallmark of OCD and other anxiety-related disorders, and this study provides an updated classification system that may improve diagnostic precision and therapeutic approaches.

Mateo-Fernández et al. identify profiles of intimate partner aggressors based on substance use and impulsivity, assessing the effectiveness of treatment programs. Given the strong link between substance use and violent behavior, this study provides important insights into the role of impulsivity in domestic violence and the potential for targeted interventions to reduce recidivism.

Broul et al. present a case report on self-amputation induced by cannabis and kratom use, highlighting extreme psychiatric manifestations of Substance Use Disorder (SUD). This rare and severe outcome underscores the need for a better understanding of the psychiatric effects of emerging psychoactive substances.

Astudillo-Reyes et al. examine causal attributions of impulsive and compulsive behaviors, shedding light on individual perceptions of these traits. Understanding how individuals interpret their own impulsive and compulsive actions may help refine cognitive-behavioral interventions aimed at modifying maladaptive thought patterns.

Pino et al. explore impulsivity as a predictor of problematic internet use in university students with disabilities, emphasizing its role in behavioral addictions. As internet addiction becomes an increasing concern, particularly among vulnerable populations, this study highlights the importance of addressing impulsivity in digital health interventions.

Cantos et al. investigate hormonal differences in perpetrators of intimate partner violence, suggesting links between aggression, impulsivity, and biological factors. This research contributes to the growing field of neurocriminology, which seeks to understand the biological underpinnings of violent behavior.

Aguilar-Yamuza et al. provide a systematic review of treatments for impulsivity and compulsivity, summarizing therapeutic approaches and their effectiveness. By comparing pharmacological and psychotherapeutic interventions, this review offers valuable guidance for clinicians seeking to tailor treatment plans to individual patients.

Yin et al. compare impulsivity levels in individuals with methamphetamine and mephedrone use disorders, with implications for treatment interventions. Understanding the specific impulsivity profiles associated with different substances can inform targeted harm reduction strategies.

Muñoz-López et al. propose correction criteria for qualitative analysis of prison populations concerning substance possession/use and gender violence. Standardizing assessment criteria in forensic settings can lead to more accurate risk assessments and better rehabilitation outcomes.

Muñoz-López et al. analyze writing patterns in incarcerated individuals with personality disorders, exploring how language reflects psychological traits. Linguistic analysis in forensic psychology is an emerging field that may provide new tools for assessing risk and treatment progress.

Roncero et al. examine gender differences in ADHD and impulsivity among patients with alcohol or alcohol and cocaine dependence, emphasizing the need for gender-specific treatment strategies. The study highlights how impulsivity manifests differently in men and women and the importance of personalized treatment approaches.

Together, these contributions enhance our understanding of impulsivity and compulsivity in psychiatric disorders, guiding future research and clinical applications in addiction, violence, and other maladaptive behaviors. By differentiating these constructs and elucidating their underlying mechanisms, this issue provides a foundation for developing more precise and effective interventions tailored to individuals with impulse control disorders. Future research should continue to explore the neurobiological basis of impulsivity and compulsivity, as well as the effectiveness of novel treatment modalities, including neuromodulation techniques and digital therapeutics. Understanding the interplay between genetic, environmental, and neurodevelopmental factors will be crucial in advancing our knowledge of these complex behavioral constructs and their role in psychiatric pathology.

